# Thioester-containing proteins regulate the Toll pathway and play a role in *Drosophila* defence against microbial pathogens and parasitoid wasps

**DOI:** 10.1186/s12915-017-0408-0

**Published:** 2017-09-05

**Authors:** Anna Dostálová, Samuel Rommelaere, Mickael Poidevin, Bruno Lemaitre

**Affiliations:** 1 Global Health Institute, School of Life Sciences, École Polytechnique Fédérale Lausanne (EPFL), CH-1015 Lausanne, Switzerland; 2 Institute for Integrative Biology of the Cell, Université Paris-Saclay, CEA, CNRS, Université Paris Sud, 1 Avenue de la Terrasse, 91198 Gif-sur-Yvette, France

**Keywords:** Innate immunity, Complement, *Beauveria*, Entomopathogenic fungus, Phagocytosis, *Drosophila*, Insect, Parasitoid wasp

## Abstract

**Background:**

Members of the thioester-containing protein (TEP) family contribute to host defence in both insects and mammals. However, their role in the immune response of *Drosophila* is elusive. In this study, we address the role of TEPs in *Drosophila* immunity by generating a mutant fly line, referred to as *TEPq* ^*Δ*^, lacking the four immune-inducible TEPs, TEP1, 2, 3 and 4.

**Results:**

Survival analyses with *TEPq* ^*Δ*^ flies reveal the importance of these proteins in defence against entomopathogenic fungi, Gram-positive bacteria and parasitoid wasps. Our results confirm that TEPs are required for efficient phagocytosis of bacteria, notably for the two Gram-positive species tested, *Staphylococcus aureus* and *Enterococcus faecalis*. Furthermore, we show that *TEPq* ^*Δ*^ flies have reduced Toll pathway activation upon microbial infection, resulting in lower expression of antimicrobial peptide genes. Epistatic analyses suggest that TEPs function upstream or independently of the serine protease ModSP at an initial stage of Toll pathway activation.

**Conclusions:**

Collectively, our study brings new insights into the role of TEPs in insect immunity. It reveals that TEPs participate in both humoral and cellular arms of immune response in *Drosophila*. In particular, it shows the importance of TEPs in defence against Gram-positive bacteria and entomopathogenic fungi, notably by promoting Toll pathway activation.

**Electronic supplementary material:**

The online version of this article (doi:10.1186/s12915-017-0408-0) contains supplementary material, which is available to authorized users.

## Background

Significant knowledge on the molecular mechanisms of innate immunity has been accumulated over the past decades using *Drosophila melanogaster* as a model organism. Upon recognition of invading microbes, several arms of innate defence are activated and coordinated in order to mount an appropriate immune response and resolve infection. *Drosophila* host defence involves both cellular and humoral modules. On the cellular side, macrophage-like blood cells (haemocytes) called plasmatocytes act as a first line of defence by phagocytosing invading bacteria. Another type of haemocytes, the large flat lamellocytes, is induced upon infestation by parasitoid wasps, contributing together with plasmatocytes to the encapsulation of these parasites [1]. Humoral mechanisms involve the fat body and haemocytes synthesising antimicrobial peptides and other immune effectors, which they release into the haemolymph. This process is regulated at the transcriptional level largely by two signalling pathways, the Imd and Toll pathways, which regulate immune genes through NF-κB transcription factors. Another mechanism of defence, specific to arthropods, is melanisation, i.e. deposition of melanin at wound sites and microbial surfaces with the concomitant release of reactive oxygen species (ROS). Melanisation is mediated by the activation of an extracellular serine protease cascade that leads to the activation of prophenoloxidase enzymes, which catalyse melanin formation. Finally, formation of clot fibres by factors released by haemocytes or the fat body has been shown to limit bacterial and nematode infection in *Drosophila* (reviewed in [1–5]). In addition to the systemic immune response that takes place in the haemolymph, epithelia that are in contact with the external environment contribute to the local immune response by producing ROS and antimicrobial peptides.

While the roles of the main immune modules and signalling pathways have been well established, the significance of many molecules induced by infection and putatively involved in defence against pathogens remains elusive. This is in part due to the fact that many immune genes belong to large families and may have overlapping functions. This redundancy hampers the assessment of their contribution to host defence unless studied at the level of the whole family [6]. Here, we analyse the function of thioester-containing proteins (TEPs) in the immune response of *Drosophila*. TEPs form a large family of immune-related proteins, which appeared early in evolution and are present in a wide range of animals including nematodes, arthropods, sea urchins, and vertebrates. The hallmark of TEPs is the presence of an intrachain thioester bond formed between a cysteine and glutamine residue in a conserved motif (CLEQ). This highly reactive site mediates covalent binding to the microbial surface by reacting with nucleophilic groups on these surfaces [7]. In mammals, TEP family members are complement factors (C3, C5, C4) and α-2-macroglobulins, molecules that play important immune functions in the complement cascade or as plasmatic protease inhibitors, respectively.

The number of genes encoding TEPs in arthropods is variable, ranging from one in some scorpions to 15 in the mosquito *Anopheles gambiae* [8]. Insect TEPs have been the focus of several studies pointing to various immune and non-immune roles. In *A. gambiae,* TEP1 has been described as an opsonin playing important roles in (1) the phagocytosis of bacteria [9], (2) the lysis of *Plasmodium* ookinetes [10], (3) the lysis and melanisation of entomopathogenic fungi [11] and (4) the removal of damaged cells during spermatogenesis [12]. TEP1 is secreted by haemocytes (or by testes) as a single-chain molecule, which is then proteolytically cleaved and stabilised by a heterodimer of the leucine-rich repeat proteins LRIM1 and APL1C [13, 14]. Upon infection, the C-terminal part of TEP1 binds to the surface of bacteria or *Plasmodium* ookinetes and promotes their phagocytosis or lysis, respectively (reviewed in [15]). Additionally, a TEP-like protein, which lacks the conserved thioester motif, has been implicated in antiviral defence in the mosquito *Aedes aegypti*. In this case, the protein does not directly bind the viral surface but interacts with a scavenger receptor-like protein recognising the virus. This viral recognition pathway leads to the induction of antimicrobial peptides which ultimately control flavivirus infection [16].

Although *Drosophila* was the first insect in which TEPs were described [17], little is known about the role TEPs play in the immune response in this genus. The *D. melanogaster* genome contains six genes encoding putative TEPs. TEP1, 2, 3 and 4 are secreted proteins expressed by epithelia, haemocytes, fat body and other tissues. Their expression is induced by various types of immune challenges in both larvae and adults. Their regulation appears to be complex, with inputs from the Toll [18, 19], Imd [18–20], JAK-STAT [17] and Mekk1 [21] pathways. Similar to other insect TEPs, the domain organisation of *Drosophila* TEPs resembles that of vertebrate α-2-macroglobulins. TEP2 has five isoforms and TEP4 has four isoforms, differing in both cases mainly in their central ’bait’ region, which contains putative target sites for proteolytic cleavage. Of interest, two of the TEP4 isoforms lack a signal peptide, suggesting that they code for intracellular proteins. Notably, TEP3 contains a putative glycosylphosphatidylinositol (GPI)-anchoring site on the C-terminus, suggesting that this protein is anchored to the plasma membrane. *Tep5* is thought to be a pseudogene, as no Tep5 transcripts are detected. *Tep6* is a more divergent member of the family. It is an essential gene, which codes for a transmembrane protein lacking the thioester motif. It is constitutively expressed in epithelia, where it is required for the formation of septate junctions [22]. Few studies have addressed the role of TEPs in *Drosophila* immunity. A study using RNA interference (RNAi) silencing in S2 cells suggested the requirement of TEP2, TEP3 and TEP6 for phagocytosis of *Escherichia coli*, *Staphylococcus aureus* and *Candida albicans*, respectively [23]. The most comprehensive study of *Drosophila* TEPs published so far used flies simultaneously deficient for the three TEPs, TEP2, TEP3 and TEP4, and found no changes in survival to infection with several species of bacteria and a fungus [24]. A recent analysis of *Drosophila* larvae infected with the entomopathogenic nematode *Heterorhabditis bacteriophora* reported increased susceptibility of *TEP3* deficient larvae to this pathogen, while *TEP2* and *TEP4* mutants were as resistant as controls [25]. It should be noted that these nematodes carry the bacterial symbiont *Photorhabdus luminescens,* which they release into the haemolymph of the infected larvae. Interestingly, a follow-up study using adult flies identified that *TEP4* mutant flies show increased resistance to this bacterium [26].

Here, we have generated a fly line lacking all the four secreted TEPs (TEP1, 2, 3 and 4), referred to as the *TEPq* ^*Δ*^ mutant throughout the study. As *Tep5* is a pseudogene and TEP6 is a constitutively expressed protein playing a role in septate junction formation, *TEPq* ^*Δ*^ flies are devoid of all the TEPs putatively involved in the immune response. Using the *TEPq* ^*Δ*^ flies, we have assessed the function of the TEP family in *Drosophila* immunity. We show that *TEPq* ^*Δ*^ flies are susceptible to entomopathogenic fungi, two species of Gram-positive bacteria and parasitoid wasps. *TEPq* ^*Δ*^ flies have an impaired capacity to phagocytose Gram-positive bacteria and a reduced Toll pathway activity upon infection by Gram-positive bacteria and fungi. Taken together, our results suggest an important role for TEPs in various modules of the *Drosophila* immune response.

## Methods

### Insect stocks

*Drosophila* stocks were maintained at 25 °C on standard fly medium [27]. Unless indicated otherwise, *w* ^*1118*^ flies were used as wild-type control. The *Relish* ^*E20*^ *(Rel* ^*E20*^ *); spaetzle* ^*rm7*^ *(spz* ^*rm7*^ *)*; *PPO1,2* ^*Δ*^; *Eater-Gal4, UAS-2xeYFP, msn9-mCherry; ModSP* ^*Δ*^ *; UAS-ModSP; psh* ^*1*^ *; GNBP3* ^*hades*^; *Lpp-Gal4*; *da-Gal4*; *hml-Gal4Δ*, *UAS-GFP* (BL30140); *UAS-secGFP* and *UAS-Bax*, *Tub-GAL80 ts*/*CyO-actin-GFP* were described previously [27–33]. Some of these insertions/mutations were combined with the *TEPq* ^*Δ*^ mutations as indicated in the figures. The following lines were used as internal controls in survival experiments: *Rel* ^*E20*^ flies which lack a functional Imd pathway and are susceptible to Gram-negative bacteria and Gram-positive bacilli, *PPO1,2* ^*Δ*^ flies which lack haemolymphatic phenoloxidase activity and are susceptible to *S. aureus* and *spz* ^*rm7*^ flies which lack a functional Toll pathway and are susceptible to other Gram-positive bacteria and fungi [27]. The *eGFP-TEP2* line (59402) was obtained from the Bloomington *Drosophila* Stock Center [34]. To generate flies lacking plasmatocytes, we crossed the *hml-Gal4Δ, UAS-GFP* to the *UAS-Bax Tub-Gal80 ts*/*CyO-actin-GFP* and kept the larvae at 18 °C [35]. The activity of the Gal4 system was activated at the late pupal stage by placing the tubes at 29 °C and keeping the flies at this temperature until infection. For overexpression of the ModSP and TEP4-GFP, the adult F1 progeny carrying Gal4 and the appropriate UAS construct was transferred from 25 °C to 29 °C 3–4 days prior to infection for optimal Gal4 efficiency. For generation of a *TEP4-GFP* overexpression construct, a full-length *Tep4* genomic DNA (*CG10363*) was amplified from BACR30H01 (CHORI) and cloned into the pTWG plasmid by Gateway technology (Invitrogen). Flies were transformed at the Fly Facility in Clermont-Ferrand, France. Parasitoid wasps of the species *Asobara tabida* and *Leptopilina boulardi* were used, and parasitisation experiments were performed as described in [27].

### Microorganism culture and infection experiments

Bacteria and fungi were cultivated and flies infected as described previously [27]. The microbial strains used and their respective optical density (OD) values of the pellet at 600 nm were as follows: the Gram-negative bacteria *Erwinia carotovora 15* (*E. carotovora*, OD 200), *Salmonella typhimurium* ATCC14028 (*S. typhimurium*, OD 10), *Enterobacter cloacae β12* (*E. cloacae*, OD 200), *Photorhabdus luminescens* (*P. luminescens*, OD 0.1) and the Gram-positive bacteria *Listeria innocua BMG449* (*L. innocua*, OD 10), *Staphylococcus aureus* (*S. aureus*, OD 0.5), *Enterococcus faecalis* (*E. faecalis*, OD 2), *Micrococcus luteus* (*M. luteus*, OD 200) and the yeast *Candida albicans* ATCC 2001 (*C. albicans*, OD 600). The fungal strains *Aspergillus fumigatus* (*A. fumigatus*), *Neurospora crassa* (*N. crassa*), *Beauveria bassiana 802* (*B. bassiana*) and *Metarhizium anisopliae KVL131* (*M. anisopliae*) were grown on malt agar plates in the dark. For septic injury with fungal spores, Petri dishes with sporulating fungi were rinsed with phosphate-buffered saline (PBS) containing 0.05% Tween-20. The recovered spores were concentrated by centrifugation, and washed 2 times with PBS and frozen until use. Infected flies were subsequently maintained at 25 °C (*S. aureus* and *E. facecalis*) or at 29 °C (all other bacteria and fungi). At least two tubes of 20 flies were used for survival experiments, and survival was scored daily. Experiments were repeated at least three times. Since natural infection by fungus tends to increase the variability between individual flies due to lack of control over the amount of spores deposited on the cuticle, we have favoured the use of septic injury when using entomopathogenic fungi. For lifespan experiments, flies were kept on normal fly medium and were flipped every 3 days. *Metarhizium anisopliae 2575-RFP* (*M. anisopliae-RFP*) spores [36] were used for phagocytosis experiments and imaging. Peptidoglycan from *E. faecalis* was purified as described previously [37] and used at a concentration of 5 mg/mL. We injected 9 nL into the thorax of female flies. Proteases of *Bacillus sp.* (Sigma) were diluted 1:1500 in PBS, and 18 nL was injected into the thorax of female flies. Frozen *B. bassiana* spores were diluted twice in PBS and inactivated by heating 1 h at 65 °C. We injected 18 nL of this preparation into the thorax of female flies.

### Wasp infestation and quantification of fly survival to wasp infestation

For wasp infections, 30 synchronised second instar wild-type or mutant larvae were deposited on the surface of a regular corn medium vial and exposed to 4 female wasps for 2 h. For survival experiments, parasitised larvae were kept at 25 °C and scored daily for pupae, flies and wasps. The difference between the number of formed pupae and the initial number of larvae was set as ‘died as larvae’. The difference between the sum of eclosed flies and wasps and the number of pupae was set as ‘died as pupae’. Data from at least three independent repeats were pooled and analysed by Pearson’s chi-square test. For imaging of lamellocytes, infested larvae were dissected 72 h after being exposed to wasps, and haemocytes were stained and counted as described in [38]. Briefly, haemocytes were allowed to adhere to glass slides, then fixed and stained with phalloidin-AF488. Mosaic images were acquired and the cell area calculated in CellProfiler. At least 2000 haemocytes were counted for each genotype.

### Melanisation assessment

Female flies were pricked in the thorax with a clean needle or a needle dipped in a *P. luminescens* solution (OD 0.1), and the level of melanisation at the wound site, estimated by the size and color of the melanin spot, was examined 3 h later.

### Phagocytosis assay

Ex vivo phagocytosis assays with larval haemocytes were performed as described in detail in [27], except that pHrodo particles were used and no trypan blue was added. Briefly, wandering L3 larvae were bled into Schneider’s Drosophila Medium containing 1 μM phenylthiourea. The medium containing haemocytes was transferred to an ultra-low attachment 96-well plate and pHrodo-labelled bacterial particles were added. The preparation was incubated at room temperature for 20 min to enable phagocytosis. For in vivo phagocytosis assays, 69 nL of pHrodo particles was injected into white prepupae using a nanoinjector (Nanoject II, World Precision Instruments, Sarasota, FL, USA). Phagocytosis was allowed to proceed for 45 min at 25 °C in a humid chamber in the dark. Subsequently, pupae were bled into PBS (pH 7.4) containing 1 μM phenylthiourea on ice. Samples were analyzed by flow cytometry with a modular Accuri C6 Flow Cytometer (BD Biosciences). pHrodo particles in solution, *w* ^*1118*^ non-injected flies, *HmlΔ > GFP* non-injected flies and *w* ^*1118*^ flies injected with pHrodo particles were used to define the gates for haemocytes and the thresholds for phagocytosed particle emission. The particles and their concentrations used were as follows: *S. aureus* (Life Technologies, 5 × 10^8^/mL ex vivo, 1.5 × 10^10^/mL in vivo), *E. coli* (Life Technologies, 1.3 × 10^10^/mL in vivo) and *E. faecalis* (labelled using the pHrodo Red Phagocytosis Particle Labeling Kit for Flow Cytometry from Life Technologies, according to manufacturer’s instructions, used at OD 0.25 ex vivo and OD 5 in vivo). Particles were sonicated and vortexed before use to achieve a homogeneous suspension. The experiment was repeated at least four times, using at least 10 larvae or 8 prepupae per genotype and experiment. For haemocyte quantification, white prepupae were bled into PBS and haemocytes counted using the Accuri cytometer from at least four batches of 10 prepupae of each genotype. Data were analysed using the Mann-Whitney test (two-sided).

### Quantitative PCR

For quantification of mRNA, whole flies were collected at indicated time points. Total fly RNA was isolated from 10–12 adult flies by TRIzol reagent and dissolved in RNase-free water. Five hundred nanograms total RNA was then reverse-transcribed in 10-μL reaction volume using PrimeScript RT (TAKARA) and a mixture of oligo-dT and random hexamer primers. For quantification of fungi, DNA was extracted from 10 infected females using a Gentra Puregene Tissue Kit (Qiagen, Hilden, Germany) according to manufacturer’s instructions. Quantitative PCR was performed on cDNA samples or on genomic DNA samples on a LightCycler 480 (Roche) in 96-well plates using the LightCycler 480 SYBR Green I Master Mix or on a LightCycler 2.0 (Roche) in capillaries using dsDNA dye SYBR Green I (Roche). Primers are listed in [27]. In addition, the following primers were used for quantification of *B. bassiana* DNA: forward 5’-GAACCTACCTATCGTTGCTTC-3’, reverse 5’-ATTCGAGGTCAACGTTCAG-3’, as reported in [39].

### Western blotting and microscopy

For Western blots, haemolymph samples were collected as follows. Twenty-five female flies were placed on a 10-μM filter of an empty Mobicol spin column (Mobitec, Goettingen, Germany), covered with glass beads and centrifuged for 20 min at 4 °C, 10,000 g into a tube containing 50 μL of PBS supplemented with complete protease inhibitor solution (Roche) and 1 mM phenylmethylsulfonyl fluoride. The protein concentration of the samples was determined by Bradford assay, and 30 μg of protein extract was separated on a 4–12% acrylamide precast Novex gel (Invitrogen) under reducing conditions and transferred to nitrocellulose membranes (Invitrogen iBlot). After blocking in 5% non-fat dry milk in PBS containing 0.1% Tween-20 for 1 h, membranes were incubated at 4 °C overnight with a mouse anti-green fluorescent protein (GFP) antibody (Roche) in a 1:1500 dilution, or a rabbit anti-lipophorin antibody (kind gift of Dr. Suzanne Eaton) in a 1:1000 dilution. Donkey anti-mouse-horseradish peroxidase (HRP) or anti-rabbit-HRP secondary antibody (Dako) in a 1:15,000 dilution was incubated for 45 min at room temperature. Bound antibody was detected using enhanced chemiluminescence (ECL, GE Healthcare) according to the manufacturer’s instructions. The blot shown is representative of two independent biological replicates. Microscope images were acquired using an Axio Imager 1 (Zeiss).

### Statistical analyses

Each experiment was repeated independently a minimum of three times (unless otherwise indicated); error bars represent the standard error of the mean of replicate experiments (unless otherwise indicated). Data were analysed using appropriate statistical tests as indicated in figure legends using the GraphPad Prism software. *P* values are represented in the figures by the following symbols: ns for *P* ≥ 0.05, * for *P* between 0.01 and 0.05, ** for *P* between 0.001 and 0.01, *** for *P* ≤ 0.001.

## Results

### TEP2 and TEP4 are found in fly haemolymph as a full-length protein and cleaved forms

**Fig. 1:**
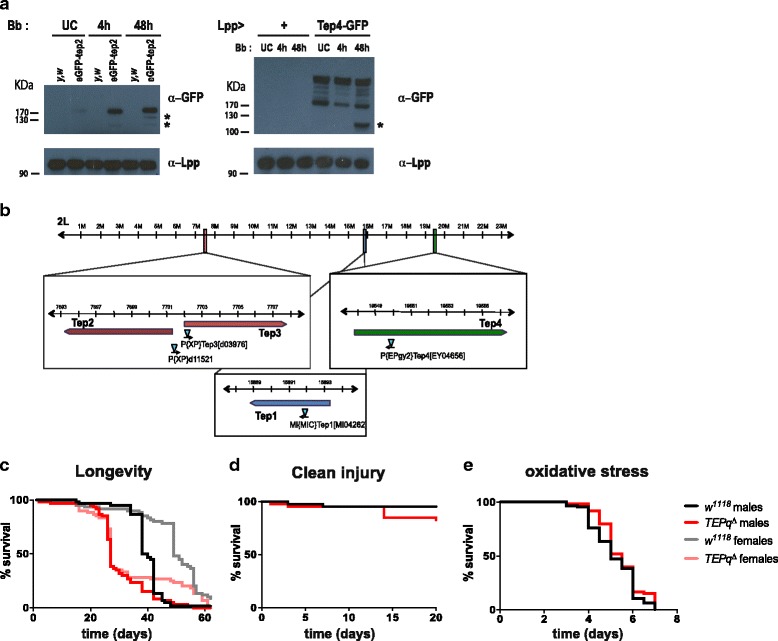
Flies devoid of inducible TEPs are viable and do not show increased susceptibility to wounding. **a** *Left panel*: eGFP-TEP2 proteins produced by an endogenously *eGFP*-tagged *TEP2* locus were detected in haemolymph samples using an anti-GFP antibody. *UC* unchallenged, *Bb* septic injury with *B. bassiana, Ctrl* haemolymph from control *y,w* flies (not expressing a GFP). Three bands corresponding in size to the full-length tagged protein and two products of proteolytic cleavage were observed (highlighted with *). *Right panel*: eGFP-TEP4 proteins produced in flies overexpressing a *TEP4-GFP* fusion using a fat body driver (genotype *UAS-TEP4-GFP/+;; Lpp-Gal4/+*). A major band with the expected size corresponding to eGFP-TEP4 was observed as well as many smaller bands. A shorter cleaved product was observed at 48 h post-infection. **b** Genomic location of the four genes encoding secreted TEPs with the position of the transposon insertions causing mutation. **c** Lifespan of unchallenged male and female flies at 25 °C. *TEPq* ^*Δ*^ flies have a shorter lifespan than the *w* ^*1118*^ controls (log-rank test, *P* < 0.001). **d** Survival to clean injury. Males were pricked in the thorax with a clean needle and kept at 25 °C. *TEPq* ^*Δ*^ flies are as resistant as the wild-type (log-rank test, *P* > 0.05). **e** Survival to oxidative stress. Flies were fed on 1.5% H_2_O_2_ in standard food and flipped on fresh medium every 2 days. *TEPq* ^*Δ*^ flies are as resistant as the wild-type (log-rank test, *P* > 0.05). **c**, **d**, **e** Shown are representative survival experiments of a minimum of two independent repeats. Forty flies minimum were used for each genotype per repeat

In order to follow the localisation and regulation of TEPs at the protein level, we made use of an engineered fly line carrying an eGFP-N-terminally tagged version of TEP2 at the endogenous locus [34]. Consistent with previous microarray data [18], Western blot on haemolymph samples using an anti-GFP antibody reveals a significant increase in the amount of TEP2-GFP at 4 h and 48 h after septic injury with the fungus *B. bassiana* (Fig. 1a, left panel). In challenged animals, we could also detected smaller sized bands, which likely correspond to proteolytically cleaved forms. In parallel, we overexpressed a *TEP4-GFP* gene fusion in the fat body (genotype *UAS-TEP4-GFP*; *Lpp-Gal4*) and analysed haemolymph samples by Western blot (Fig. 1a, right panel). A major band with the expected size corresponding to full-length eGFP-TEP4 was observed, as well as several smaller bands. Interestingly, a shorter cleaved product was observed at 48 h post-infection. These experiments are consistent with the notion that TEP2 and TEP4 are secreted into the haemolymph and undergo proteolytic cleavage upon infection, similarly to what was reported for *A. gambiae* TEP1 [9].

### Flies devoid of all inducible TEPs are viable and do not show increased susceptibility to wounding

In order to study the role of inducible TEPs in the immune response of *Drosophila*, we created a compound knockout fly line lacking all the four genes encoding secreted TEPs (*Tep1, 2, 3, 4*). To this end, we made use of the previously described [24] knockout lines for Tep2, 3 and 4 (*Tep2,3* ^*Δ*^ created by FLP-mediated recombination between *XP* elements inserted in the two genes and an *EY04656* line carrying a *P* element inserted at the initiation codon of the *Tep4* gene). We recombined the latter line with a knockout of *Tep1* (*MI04262* carrying a MiMiC element insertion in the second exon of the *Tep1* gene) (Fig. 1b, Additional file 1: Figure S1A). Double knockout (*Tep2,3* ^*Δ*^ and *Tep1,4* ^*Δ*^) lines were backcrossed five times into *w* ^*1118*^ to homogenise the genetic background and then recombined. We refer to the resulting fly line lacking all the four inducible TEPs as *TEPq* ^*Δ*^ (i.e. quadruple TEP knockout) (Additional file 1: Figure S1A). These flies, despite showing a significantly shorter lifespan under standard laboratory conditions as compared to wild-type flies (Fig. 1c) and a lower viability (Additional file 1: Figure S1B), do not exhibit any overt developmental or behavioural defects. The expression of *Drosophila Tep1* and *Tep2* have been shown to be regulated by the JAK-STAT pathway, which is involved in tissue regeneration and wound healing [17, 40, 41]. This prompted us to study the impact of *TEPq* ^*Δ*^ in wound healing and JAK-STAT pathway activation. *TEPq* ^*Δ*^ flies did not show an increased sensitivity to wounding (Fig. 1d). We did not detect any overt defect in the activity of the JAK-STAT pathway, as illustrated by normal expression of the JAK-STAT pathway target gene *Socs36E* (Additional file 1: Figure S1C). To uncover a possible role of TEP in the response to stress, we also tested their resistance to oxidative stress by exposing wild-type and *TEPq* to continuous feeding of 1.5% hydrogen peroxide. Figure 1e shows that the *TEPq* ^*Δ*^ mutant survived as well as the control *w* ^*1118*^ flies and thus did not show any increased sensitivity to ROS under these experimental conditions (Fig. 1e). Hereafter, we focus on the role of TEPs in the defence against microbial pathogens and parasites.

### Flies devoid of secreted TEPs are susceptible to Gram-positive bacteria, entomopathogenic fungi and parasitoid wasps

**Fig. 2:**
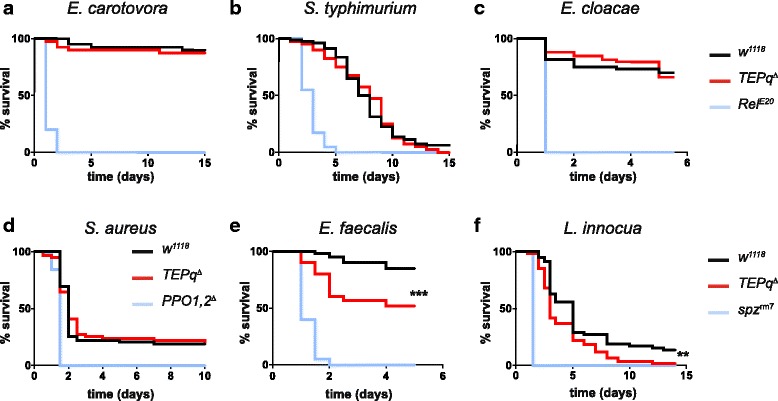
Survival to systemic bacterial infection. Male flies were pricked in the thorax with a needle dipped in a concentrated bacterial culture. Data were analyzed by log-rank test. Shown are representative experiments of a minimum of two independent repeats (three where a difference from the control flies was observed). *x*-axis: time post-infection in days; *y*-axis: percentage of living flies. **a**–**d** Survival to septic injury with Gram-negative bacteria (*E. carotovora*, *S. typhimurium* and *E. cloacae*) and the Gram-positive bacterium *S. aureus*. No statistically significant difference was observed between *TEPq* ^*Δ*^ and wild-type (*w* ^*1118*^) flies. **e**, **f** Survival to septic injury with Gram-positive bacteria *E. faecalis* and *L. innocua*. Statistically significant differences were observed between *TEPq* ^*Δ*^ and wild-type (*w* ^*1118*^) flies (*P* < 0.001 for *E. faecalis* and *P* = 0.00172 for *L. innocua*)

**Fig. 3:**
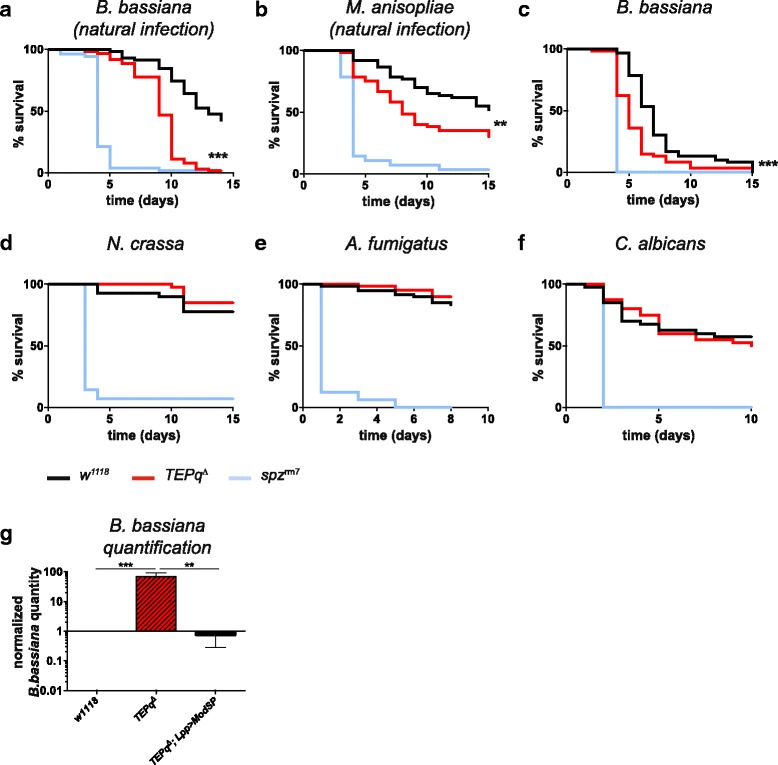
Survival to fungal infection. Male flies were either covered with spores (**a**, **b**, labelled as natural infection) or pricked in the thorax with a needle dipped in a concentrated fungal spore suspension (**c**–**f**). Data were analyzed by log-rank test. Shown are representative experiments of a minimum of two independent repeats (three where a difference from the control flies was observed). *x*-axis: time post-infection in days; *y*-axis: percentage of living flies. **a**–**c** Statistically significant differences were observed between *TEPq* ^*Δ*^ and wild-type (*w* ^*1118*^) flies (*P* < 0.001 for *B. bassiana*, both natural infection and pricking; *P* = 0.0039 for *M. anisopliae*). **d**–**f** No statistically significant difference was observed between the *TEPq* ^*Δ*^ and the wild-type (*w* ^*1118*^) flies in the case of *N. crassa*, *A. fumigatus* and *C. albicans* infection by septic injury. **g** Quantification of *B. bassiana* DNA 3 days post-infection normalised to the host *RpL32* DNA. Values represent the mean ± standard error (*SE*) of three independent experiments and were analysed using Mann-Whitney test (two-sided). The quantity of fungal DNA is significantly elevated in the *TEPq* ^*Δ*^ flies as compared to the control *w* ^*1118*^ line (*P* < 0.001). Overactivation of the Toll pathway in *TEPq* ^*Δ*^ flies by overexpressing *ModSP* rescues the increased fungal growth caused by the absence of *TEPq* ^*Δ*^ (*P* = 0.005)

**Fig. 4:**
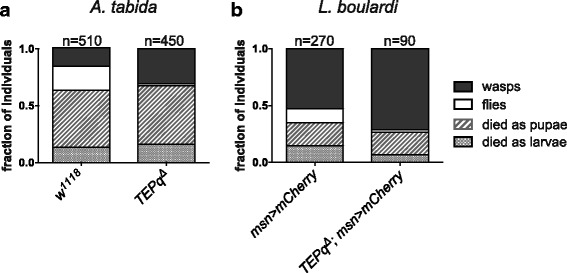
Survival to parasitoid wasps. Second instar larvae were exposed to female parasitoid wasps, and the emergence of wasps (*black boxes*) and flies (*white boxes*) was monitored. Of note, a significant fraction of infested animals die as larvae and pupae (*dashed boxes*). We observed a significant difference in the outcome of infection between the *TEPq* ^*Δ*^ and the wild-type flies (**a** *A. tabida*, chi-square = 97.59, df = 3, *P* < 0.001; **b** *L. boulardi,* chi-square = 14.81, df = 3, *P* = 0.02). In the case of *L. boulardi* infections, flies carrying a lamellocyte marker (*misshapen-Gal4, UAS-mCherry*) were used to monitor lamellocyte differentiation. Results are represented as a sum of a minimum of three independent experiments. Statistical significance was calculated using Pearson’s chi-square test

In order to test their resistance to microbial infection, we challenged the *TEPq* ^*Δ*^ mutant flies with a panel of bacterial and fungal pathogens. We compared their survival to that of the *w* ^*1118*^ line (referred to as the wild type) used to isogenise the *TEP* mutations. As highly susceptible controls, we used immune deficient flies lacking either a functional Imd (*Rel* ^*E20*^ *)* or Toll pathway *(spz* ^*rm7*^), or lacking haemolymph phenoloxidase activity (*PPO1,2* ^*Δ*^ *)* [27]. We observed that *TEPq* ^*Δ*^ flies were as resistant as the wild type to systemic infection with all the Gram-negative bacteria species tested (*E. carotovora 15*, *S. typhimurium* and *E. cloacae,* Fig. 2a, b, c). Similarly, they did not show an increased susceptibility to the Gram-positive bacterium *S. aureus* (Fig. 2d). However, we observed a decreased resistance to the other two Gram-positive bacteria tested: *E. faecalis* and *L. innocua* (Fig. 2e, f). This discrepancy may be due to the fact that immunity to the latter two Gram-positive bacteria relies primarily on a functional Toll pathway, while immunity to *S. aureus* relies primarily on phagocytosis and melanisation [35, 42]. Interestingly, flies lacking TEPs showed an increased susceptibility to two entomopathogenic fungi, *B. bassiana* and *M. anisopliae* (Fig. 3a, b). This effect was observed when fungal spores were deposited on the cuticle (referred to as ‘natural infection’, Fig. 3a, b). *TEPq* ^*Δ*^ flies also showed increased susceptibility when spores of *B. bassiana* were directly introduced into the body cavity by pricking with a needle (Fig. 3c). However, flies lacking TEPs were as resistant as the wild type upon septic injury with three other fungi, *N. crassa*, *A. fumigatus,* an opportunistic fungus, and *C. albicans*, a yeast (Fig. 3d–f). Thus, the increased susceptibility to fungal infection seems to be restricted to entomopathogenic species capable of naturally establishing a systemic infection in *D. melanogaster* [43]. We then analysed whether the lower survival of the *TEPq* ^*Δ*^ flies was associated with higher pathogen loads. To this end, we infected wild-type and *TEPq* ^*Δ*^ flies by pricking with *B. bassiana* spores and quantified by qPCR the fungal DNA present 3 days after treatment. Consistent with the increased susceptibility, we did observe a markedly higher quantity of fungal DNA in *TEPq* ^*Δ*^ flies as compared to the wild type (Fig. 3g). These results indicate that TEPs contribute to elimination of the fungus or constrain its growth. We next assessed the role of TEPs in the defence against parasitoid wasps. We exposed second instar larvae to females of two parasitoid wasp species, *A. tabida* and *L. boulardi*. Lamellocyte differentiation, encapsulation and melanisation of wasp eggs were observed even in the absence of TEPs. The average ratio of lamellocytes to the whole haemocyte population in larvae 3 days after exposure to *A. tabida* was similar in the *TEPq* ^*Δ*^ (13.5 ± 8%) and the wild type (13.8 ± 6%). However, with both wasp species, there was a marked increase in the number of emerging wasps in the *TEPq* ^*Δ*^ as compared to wild-type wasp-infested larvae (Fig. 4). This points to a role of TEPs at a specific stage of the encapsulation process, a hypothesis we did not explore further in this study.

### Secreted TEPs are required for efficient phagocytosis of *E. faecalis* and *S. aureus* but not *E. coli*

**Fig. 5:**
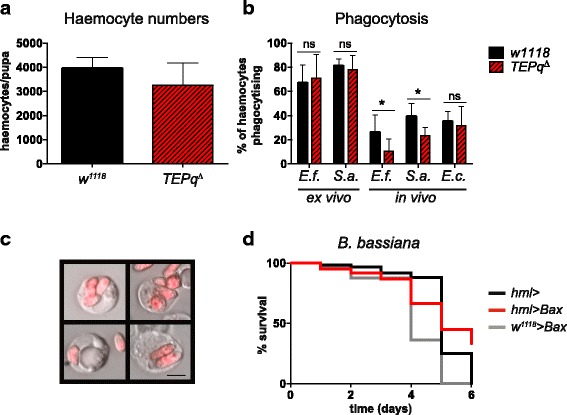
Phagocytosis of bacteria. **a** The number of haemocytes in prepupae in the *TEPq* ^*Δ*^ and wild-type *w* ^*1118*^ line. No statistically significant difference was observed (Mann-Whitney test, two-sided). **b** Phagocytosis of bacterial particles assessed by ex vivo and in vivo phagocytosis assays. Significantly lower rates of phagocytosis of the Gram-positive bacteria *E. faecalis* (*P* = 0.032) and *S. aureus* (*P* = 0.015) were detected in the *TEPq* ^*Δ*^ prepupae in the in vivo assay. No statistically significant differences were observed in phagocytosis of *E. coli* in vivo or *E. faecalis* or *S. aureus* ex vivo. Data were pooled from at least four independent experiments and analysed by Mann-Whitney test (two-sided). **c** Representative images of haemocytes of third instar larvae with internalised spores of *M. anisopliae 2575-RFP* after incubation in vitro. Scale bar represents 5 μm. **d** Flies with reduced number of plasmatocytes (*hmlΔ-Gal4* > *UAS-Bax*) do not show an increased susceptibility to septic injury with *B. bassiana*. Data were analyzed by log-rank test. Shown is a representative experiment of two independent repeats

The increased susceptibility of flies lacking TEPs to a number of pathogens led us to examine in which defence mechanisms they are involved. Given that TEPs were previously described as opsonins [23], we started by assessing their role in the phagocytosis of bacteria. Larvae and adults differ in their immune response with respect to haemocytes. Due to the low number of haemocytes in adult flies and technical difficulties in standardised quantification of phagocytosis rates in adults, we performed the experiments at larval and pupal stages. We first quantified the number of circulating haemocytes in white prepupae (i.e. shortly after puparium formation). We did not observe any consistent differences in the number of haemocytes between the *TEPq* ^*Δ*^ and wild-type pupae (Fig. 5a). We then assessed the ability of *TEPq* ^*Δ*^ haemocytes to phagocytose bacterial particles. Larval haemocytes were bled and diluted in medium containing bacterial particles and phagocytosis was allowed to proceed in vitro as described in [38]. Under these ex vivo conditions, haemocytes derived from *TEPq* ^*Δ*^ larvae were as efficient in phagocytosis of both *S. aureus* and *E. faecalis* particles as the wild type (Fig. 5b). Given that TEPs1, 2 and 4 are secreted proteins and are produced by various organs including the fat body, the lack of an observable phenotype in the ex vivo phagocytosis assay could be due to the fact that haemolymph (and the TEPs it contains) was diluted. We therefore performed an in vivo assay in order to assess the role of TEPs in phagocytosis. To this end, we injected bacterial particles into white prepupae and let the phagocytosis proceed in vivo as described in the ‘Methods’ section. Interestingly, the in vivo assay revealed a significant decrease in the rate of phagocytosis of particles of two Gram-positive bacterial species, *E. faecalis* and *S. aureus*, in the *TEPq* ^*Δ*^ line (Fig. 5b). In contrast, the absence of TEPs did not impair the phagocytosis of particles of the Gram-negative bacterium *E. coli* (Fig. 5b). Overall, the phagocytosis defects were in agreement with the higher susceptibility of the *TEPq* ^*Δ*^ flies to some Gram-positive but not to Gram-negative bacteria. However, note that, even though *TEPq* ^*Δ*^ flies are impaired in phagocytosing *S. aureus* particles, they survived as well as the wild type to *S. aureus* infection.

### Resistance of flies to *B. bassiana* does not rely on plasmatocytes

Since we observed an increased fungal burden in *TEPq* ^*Δ*^ flies and their high susceptibility to fungal infection, we speculated that TEPs might also function as opsonins promoting phagocytosis of entomopathogenic fungi, similarly to what we described above for Gram-positive bacteria. Previous studies done in *M. anisopliae* suggest that phagocytosis and encapsulation by haemocytes contribute to the defence against fungal pathogens in insects [1, 43, 44]. However, to our knowledge, there is no study directly addressing the role of haemocytes in host defence against entomopathogenic fungi in *Drosophila*. Thus, we first set out to assess the importance of phagocytosis in defence against these pathogens. We observed that larval haemocytes were capable of internalising *M. anisopliae* spores when exposed to them in vitro (Fig. 5c). However, the percentage of cells that had phagocytosed a spore after 40 min of exposure remained under 1%. In addition, we did not observe any phagocytosing cells when injecting fungal spores into white prepupae (data not shown). To further address the relevance of phagocytes in the resistance to fungal infection, we generated flies lacking most plasmatocytes by overexpressing the pro-apoptotic gene *Bax* with the plasmatocyte driver *hemolectin-Gal4* [35], and subsequently infected them with *B. bassiana* spores by pricking. Figure 5d shows that these ‘Phagoless’ flies survived as well as the wild type (Fig. 5d), suggesting no role or a negligible role of plasmatocytes in the protection against fungi under the tested conditions. We thus concluded that the observed susceptibility of *TEPq* ^*Δ*^ flies to *B. bassiana* and *M. anisopliae* was unlikely to result from a possible defect in phagocytosis.

### Secreted TEPs promote Toll pathway activation

**Fig. 6:**
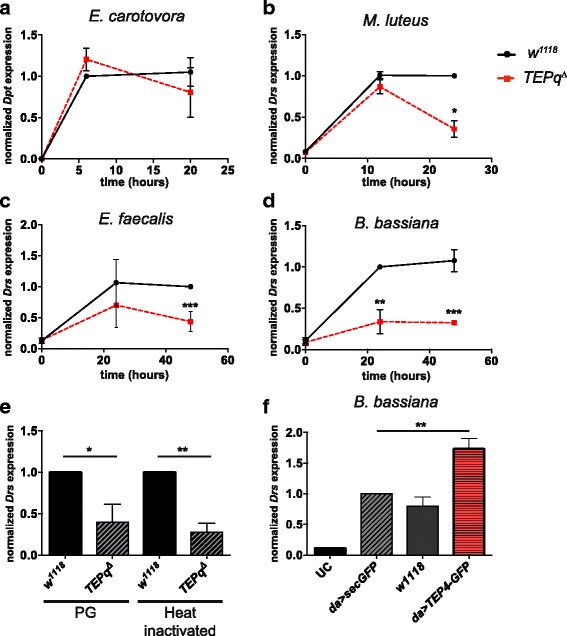
Toll and Imd pathway induction in the *TEPq* ^*Δ*^ mutant. Expression of antimicrobial peptide genes normalised to ribosomal protein gene *RpL32* after septic injury. **a** Induction of *Diptericin* (*Dpt*, Imd pathway read-out) in response to septic injury with *E. carotovora. TEPq* ^*Δ*^ flies show a wild-type level of induction of *Diptericin*. **b** Induction of *Drosomycin* (*Drs*; Toll pathway read-out) in response to septic injury with *M. luteus. TEPq* ^*Δ*^ flies show a significantly lower level of *Drs* expression 24 h post-infection (*P* = 0.013). **c** *TEPq* ^*Δ*^ flies show a significantly lower level of *Drs* expression 24 h post-infection with *E. faecalis* (*P* < 0.001 at 48 h post-infection). **d** *TEPq* ^*Δ*^ flies show a reduced *Dr*s expression after septic injury with *B. bassiana* (*P* = 0.005 at 24 h post-infection and *P* < 0.001 at 48 h post-infection). **e** *TEPq* ^*Δ*^ flies show reduced *Drs* expression in response to the injection of purified *E. faecalis* peptidoglycan (*PG*; measured 16 h post-injection, *P* = 0.0286, Mann-Whitney test, two-sided) and heat inactivated spores of *B. bassiana* (*Heat inactivated*; measured 16 h post-injection, *P* = 0.0079, Mann-Whitney test, two-sided). **f** *Drosomycin* expression 24 h after challenge with *B. bassiana* is enhanced in *TEP4-GFP* overexpressing flies (*P* = 0.0079). *UC* unchallenged; *Sec-GFP*, flies overexpressing a secreted form of GFP were used as control. Data were analysed using *t* test comparing the values in *TEPq* ^*Δ*^ flies to wild-type *w* ^*1118*^ flies. Values represent the mean ± SE of at least two independent experiments

Besides phagocytosis, hallmarks of systemic immunity in *Drosophila* are melanisation, clotting and production of antimicrobial peptides by the fat body. Thus, we assessed the contribution of TEPs to these three processes. There was no striking difference in melanin deposition at the wound site in adults when comparing *TEPq* ^*Δ*^ and wild-type animals (Additional file 1: Figure S1D), indicating that TEPs are dispensable for this process. Similarly, formation of clot fibres in the haemolymph, as assessed by the hanging drop method [45], was comparable to that of wild-type larvae (data not shown). We next investigated the role of TEPs in the regulation of the Toll and Imd pathways, using antimicrobial peptide genes as read-outs [1]. We measured expression of the Imd target gene *Diptericin (Dpt)* in response to infection with the Gram-negative bacterium *E. carotovora 15*, and did not find any differences between *TEPq* ^*Δ*^ and wild-type flies (Fig. 6a). This result is consistent with our finding that flies lacking TEPs do not show any increased susceptibility to Gram-negative bacteria. We then analysed the expression of the Toll target gene *Drosomycin (Drs)* upon septic injury with the Gram-positive bacteria *M. luteus* and *E. faecalis* and the fungus *B. bassiana*. Strikingly, *Drs* induction was lower in *TEPq* ^*Δ*^ flies compared to wild-type flies in these conditions (Fig. 6b–d). Similarly, the expression of *Drs* was weaker in response to the injection of purified peptidoglycan from *E. faecalis* or injection of heat-inactivated fungi (Fig. 6e). Conversely, overexpression of the *TEP4-GFP* gene fusion using the ubiquitous driver *daughterless* increased the levels of *Drs* expression after septic injury with *B. bassiana* (Fig. 6f). Collectively, these results reveal that TEPs contribute to activating the Toll pathway in response to Gram-positive and fungal infection, consistent with the increased susceptibility of *TEPq* ^*Δ*^ flies to these germs.

### TEPs function upstream or independently of ModSP

**Fig. 7:**
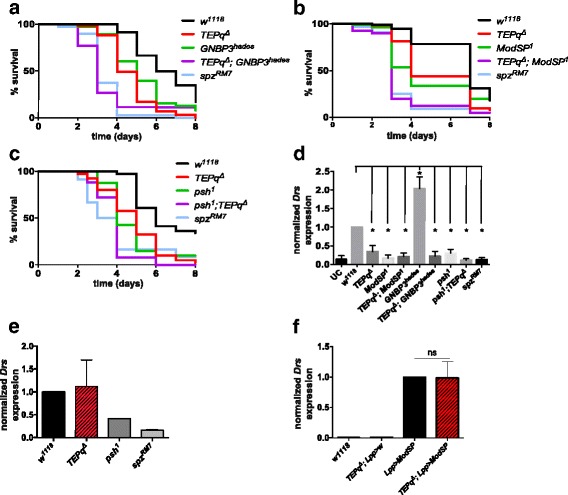
Role of TEPs in regulation of the Toll pathway. **a**–**c** Male flies were pricked in the thorax with a needle dipped in a concentrated suspension of *B. bassiana* spores. Data were analyzed by log-rank test. Each panel shows a representative experiment of a minimum of three independent repeats. *x*-axis: time post-infection in days; *y*-axis: percentage of living flies. **a** *TEPq* ^*Δ*^ *, GNBP3* ^*hades*^ double mutant flies are highly susceptible to *B. bassiana* as compared to the *w* ^*1118*^ control line (*P* < 0.001) with a survival curve comparable to that of *spz* ^*rm7*^ flies. *TEPq* ^*Δ*^ or *GNBP3* ^*hades*^ single mutant flies show a milder phenotype (*P* = 0.004 and *P* < 0.001, respectively, as compared to the double mutants). **b** *TEPq* ^*Δ*^ *, ModSP* ^*1*^ double mutant flies are highly susceptible to *B. bassiana* as compared to the *w* ^*1118*^ control line (*P* < 0.001) with a survival curve comparable to that of *spz* ^*rm7*^ flies. *TEPq* ^*Δ*^ or *ModSP* ^*1*^ simple mutant flies show a milder phenotype (*P* < 0.001 for both as compared to the double mutant). **c** *psh* ^*1*^ *, TEPq* ^*Δ*^ double mutant flies show an increased susceptibility to *B. bassiana* compared to the *w* ^*1118*^ wild-type flies (*P* < 0.001). The survival curve is comparable to that of *spz* ^*rm7*^ flies. *TEPq* ^*Δ*^ or *psh* ^*1*^ single mutant flies show a milder phenotype (*P* = 0.002 and *P* = 0.005, respectively), as compared to the double mutants. **d** Female flies were pricked in the thorax with a needle dipped in a concentrated suspension of *B. bassiana* spores, and the expression of *Drs* was measured 24 h post-infection. Values represent the mean ± SE of three independent experiments and were analysed using the Mann-Whitney test (two-sided). *Drs* expression for all the genotypes tested except for *GNBP3* ^*Δ*^ was significantly lower than in the wild-type *w* ^*1118*^ flies, as indicated by asterisks in the chart (0.01 < *P* < 0.03 for all cases). There were no statistically significant differences between *TEPq* ^*Δ*^ flies and other compound knockouts (i.e. *TEPq* ^*Δ*^ *, GNBP3* ^*hades*^ or *TEPq* ^*Δ*^ *, ModSP* ^*1*^ or *psh* ^*1*^ *, TEPq* ^*Δ*^.) **e** *Drs* expression in response to injection of purified proteases of *B. subtilis*. No statistically significant differences were observed between the *TEPq* ^*Δ*^ and the wild type *w* ^*1118*^. **f** *Drs* expression in unchallenged male flies of the indicated genotypes. Values represent the mean ± SE of three independent experiments and were analysed using the Mann-Whitney test (two-sided). Overexpression of *ModSP* induces *Drs* expression to the same levels in the presence or absence of TEPs

Previous studies support the existence of two serine protease cascades that link microbial recognition to the activation of the Toll receptor ligand Spz: the pattern-recognition receptors (PRRs) and the Persephone (Psh) pathways [29, 46]. The PRR pathway is initiated upon auto-activation of a serine protease, ModSP, after the sensing of peptidoglycan by a complex of peptidoglycan recognition protein (PGRP)-SA and Gram-negative bacteria-binding protein 1 (GNBP1) or the recognition of ß-1,3-glucan by GNBP3 [47]. Certain bacterial and fungal species can activate the Toll pathway independently of PRRs through the Psh pathway. This mode of activation is initiated upon direct detection of proteases released by pathogens. It has been suggested that the presence of *B. bassiana* is recognised both by GNBP3, which binds to glucans of the cell wall, and by the activation of the Psh pathway by the fungal protease PR1 [28, 29]. In order to further elucidate how TEPs affect the Toll pathway, we recombined the *TEPq* ^*Δ*^ compound knockout with either a mutation in *GNPB3* (*GNBP3* ^*hades*^), *ModSP* (*ModSP* ^*1*^) or *psh* (*psh* ^*1*^). We then followed *Drs* expression as well as survival after pricking with the entomopathogenic fungus *B. bassiana. TEPq* ^*Δ*^ *, GNBP3* ^*hades*^ combined mutant flies were highly susceptible to *B. bassiana* compared to single mutants, in fact not differing in their survival from flies carrying a null mutation in *spz* (Fig. 7a). Similarly, *TEPq* ^*Δ*^ *ModSP* ^*1*^ combined mutant flies showed an increased susceptibility to *B. bassiana* compared to *TEPq* ^*Δ*^ or *ModSP* ^*1*^ single mutant flies (Fig. 7b). A possible explanation of these results would be that TEPs function not in the PRR arm, but in the Psh arm of the Toll pathway. In this case, we would expect flies lacking both *TEPs* and *psh* to display a level of Toll activation similar to *psh* ^*1*^ alone. In contradiction with this hypothesis, we observed that *psh* ^*1*^ *, TEPq* ^*Δ*^ were less resistant to *B. bassiana* than either *TEPq* ^*Δ*^ or *psh* ^*1*^ mutants (Fig. 7c). Analysis of *Drs* expression showed that flies carrying *TEPq* ^*Δ*^ in combination with *GNBP3* ^*hades*^ or *psh* ^*1*^ have slightly lower activation of the Toll pathway compared to single mutants, but the differences were too small to reach significance (Fig. 7d)*.* The slight differences observed in our survival and *Drs* analyses did not allow us to reach a definitive conclusion on the position of TEPs in the two branches. Interestingly, upon injection of proteases from *Bacillus subtilis*, a stimulus that activates only the Psh branch [46], *Drs* was induced in *TEPq* ^*Δ*^ flies to a level comparable to that of the wild type (Fig. 7e). These data indicate that TEPs are not mandatory for the activation of the Psh pathway. The lower Toll activation observed in *TEPq* ^*Δ*^ flies upon injection of peptidoglycan, a stimulus inducing only the PRR pathway, supports a role of TEPs in this pathway, possibly at an early step by facilitating the recognition of pathogens by PRRs. To test this hypothesis, we analysed whether *TEPq* ^*Δ*^ mutations can block Toll activity provoked by the overexpression of ModSP. Figure 7f shows that *TEPq* ^*Δ*^ deficiency did not affect Toll pathway activation upon *ModSP* overexpression, thus further confirming that TEPs do not function downstream of this apical serine protease. Overexpression of *ModSP* also efficiently slowed fungal proliferation in the *TEPq* ^*Δ*^ mutant (Fig. 3g). Altogether, these results suggest that TEPs function upstream of ModSP at an early step of Toll pathway activation.

## Discussion

In this study, we investigated the role of TEPs in the immune response of *Drosophila*. In line with previously published transcriptomic data [24], we confirm the upregulation of TEP2 in response to infection. We also detected several forms of TEP2 and TEP4 in haemolymph samples: the full-sized protein and smaller forms, which are likely the result of proteolytic cleavage. This suggests that TEPs are secreted as single-chain proteins and then cleaved by an extracellular protease. Our results on the expression and cleavage of TEPs are reminiscent of what is described for TEP1 in mosquitoes [9, 15]. Interestingly, *Drosophila* lacks orthologues of the LRR proteins that have been shown to stabilise a cleaved form of TEP1 in *A. gambiae* [13, 14]. Further studies are required to unravel the mechanisms involved in the proteolytic processing and regulation of TEPs in *Drosophila* and to identify potential stabilising partners.

Use of a compound mutant, *TEPq* ^*Δ*^, which lacks all inducible TEPs, reveals the importance of TEPs in defence against various microbes and parasites. More specifically, we found that *TEPq* ^*Δ*^ flies display increased susceptibility to two Gram-positive bacterial species, *E. faecalis* and *L. innocua,* but not to *S. aureus* or any of the three Gram-negative bacteria tested (*E. carotovora*, *S. typhimurium* and *E. cloacae*). Our study does not exclude a role of TEPs against specific Gram-negative bacteria, such as *Porphyromonas* and *Photorhabdus*, as suggested by previous studies [26, 48]. The differences in susceptibility observed within the Gram-positive bacterium clade is likely due to the role of TEPs in Toll pathway activation, which has been shown to control *E. faecalis* and *L. innocua* but not *S. aureus* [35, 42]. Our study also reveals a decreased resistance of *TEPq* ^*Δ*^ flies to two entomopathogenic fungi, namely *B. bassiana* and *M. anisopliae*. This effect was independent of the route of infection (deposition of spores on the cuticle or pricking with a needle covered with spores), and it was associated with a higher fungal growth rate. Interestingly, the *TEPq* ^*Δ*^ mutant is as resistant as the wild type to the other three fungal species tested (*N. crassa*, *A. fumigatus* and *C. albicans*). While we cannot explain these differences, our results point to a certain level of specificity in the microbe targeted by TEPs. Interestingly, TEP1 and to a lesser extent TEP2 have been shown to be rapidly evolving genes under positive selection in *Drosophila* [49]. This pattern is consistent with host-parasite coevolution, which is most likely to occur when host and parasite proteins interact [50]. Thus, the susceptibility of *TEPq* ^*Δ*^ flies to specific pathogens within a clade is likely due to the direct interaction of TEPs with specific components of microbial surfaces, which might evolve under the pressure of the immune system.

Our genetic analysis indicates that *Drosophila* TEPs have multiple functions contributing to both cellular and humoral immunity. In line with an in vitro study conducted in S2 cells [23], we show that TEPs are required for phagocytosis of bacteria. However, in contrast to the above-mentioned results, which implicated TEP2 in the phagocytosis of *E. coli*, a role of TEPs in phagocytosis was detected only when using an in vivo assay and was restricted to certain Gram-positive bacteria. Strikingly, we were not able to detect any defect at all using an ex vivo phagocytosis assay. The fact that TEPs contribute to phagocytosis in the in vivo (prepupae) but not ex vivo assay (haemocytes supplemented with S2 medium) is consistent with a role of TEPs as opsonins. A contribution of phagocytosis to antifungal immunity in *Drosophila* has not yet been fully established [43], but our results suggest no role or only a negligible role of phagocytes in the protection against the entomopathogenic fungus *B. bassiana*, at least under the conditions tested.

Melanisation is an important immune module that contributes to survival to Gram-positive bacteria, fungi and parasites [38]. TEPs have been described to promote melanisation of bacteria and of fungal hyphae in mosquitoes [11, 51]. A recent study suggested a role of *Tep4* in the melanisation response upon pricking with bacteria of the genus *Photorhabdus* [26]. In contrast, we did not find any signs of a compromised or reinforced melanisation of wounds in flies devoid of the four TEPs upon clean injury or upon septic injury with *P. luminescens* (Additional file 1: Figure S1E). We also did not observe any melanisation of bacteria or of fungal spores or hyphae in vivo. Nevertheless, we cannot exclude a role of TEPs in the melanisation to specific pathogen strains or in certain infectious contexts that were not analysed in the present study.

In response to parasite infestation, lamellocytes differentiate from haemocyte progenitors in the lymph gland or directly from plasmatocytes present in the periphery to form a capsule around the pathogen [52–54]. Given that TEP4 is expressed in the lymph gland [55], we expected a role of TEPs in the encapsulation process. After infesting larvae with two wasps from two distinct genera, we observed a higher rate of successfully parasitised *TEPq* ^*Δ*^ larvae (e.g. giving rise to wasps rather than flies) compared to the wild type. This effect could in part be due to the higher lethality of *TEPq* ^*Δ*^ flies. We did not find any contribution of TEPs in the encapsulation of wasp eggs by plasmatocytes or in the production of lamellocytes. It should be noted that wasp larvae escape from capsules that appear to be fully formed and melanised both in the *TEPq* ^*Δ*^ and the wild-type line. In conclusion, the results of our experiments suggest a role for TEPs against parasitoid wasps, which has yet to be mechanistically characterised.

One of the most surprising results was the observation that TEPs contribute to Toll pathway activation. While Imd pathway activation was fully comparable to that observed in the wild-type control line, *TEPq* ^*Δ*^ flies showed a defect in Toll pathway activation in response to Gram-positive bacteria and fungi. This effect was particularly strong in *B. bassiana*-infected flies. The significant level of *Drosomycin* expression observed in *TEPq* ^*Δ*^ flies in response to Gram-positive bacteria indicates that TEPs are not mandatory for Toll pathway activation as canonical Toll pathway components but rather promote its full activation. Injection experiments using purified peptidoglycan or microbial proteases show that TEPs are likely involved in the recognition of microbes per se rather than the detection of their secreted virulence factors. One possibility is that they promote microbial recognition and activation of the protease ModSP in parallel to or upstream of pattern-recognition receptors. In this light, it is interesting to note that null mutations in *ModSP* or *GNPB3* affect Toll pathway activation in a different way. While the level of *Drosomycin* expression upon *B. bassiana* infection is reduced in the *ModSP* ^*1*^ knockout, it is not affected (or even enhanced) by the *GNBP3* mutation ([29, 47] and this study). This suggests the existence of a yet uncharacterised molecule promoting the activation of ModSP at least in response to entomopathogenic fungi. Studies done in the Lepidopteran *Manduca sexta* and the beetle *Tenebrio molitor* indicate that the protease ModSP likely physically interacts with GNBP3 upon recognition of fungi [56, 57]*.* Interestingly, ModSP contains complement control protein domains (CCPs). In vertebrates, these domains mediate interaction of regulatory proteins, including proteases, with the components C3b and C4b of the complement cascade [58]. It is tempting to speculate that a complex involving TEPs and ModSP is assembled on the surface of pathogens leading to the full activation of Toll signalling. However, the additive phenotype in terms of resistance to *B. bassiana* observed when combining the *TEPq* ^*Δ*^ and the *ModSP* ^*1*^ mutations suggests that TEPs do not function exclusively upstream of ModSP.

One limitation of studies involving compound mutants is a possible effect of the genetic background and the difficulty in performing rescue experiments. We have made considerable efforts to reduce the influence of the genetic background by isogenising the TEP mutations with the wild-type *w* ^*1118*^ background before starting the experiments. In support of our results, a significantly increased susceptibility to *B. bassiana* was also seen in *TEPq* flies generated in another background as compared to two different wild-type flies (Additional file 2: Figure S2A). We were also able to enhance *Drosomycin* expression upon fungal infection by overexpressing one of the TEPs. Finally, we observed a modest contribution of *Tep1* single mutant and *TEP1, 4* and *TEP2,3* double mutants to survival to septic injury with *B. bassiana* (Additional file 2: Figure S2B). The much higher susceptibility of *TEPq* ^*Δ*^ flies to *B. bassiana* compared to single or double mutants suggests that TEPs contribute additively to survival and that the function of individual TEPs is partially masked by the contribution of the others. Further studies are needed to clarify the role of individual TEPs in the immune defence or elucidate in detail the molecular mechanisms of action of these proteins in *Drosophila.* We cannot exclude an important role of TEPs in epithelial immunity against foodborne Gram-negative pathogens, as TEPs are also induced in the gut [59]. Preliminary observations suggest that the *TEPq* ^*Δ*^ mutant exhibits higher susceptibility to oral infection with *Pseudomonas entomophila* (Additional file 4: Table S2). 

## Conclusions

Collectively, our study brings new insights into the function of TEPs in the immune response of *Drosophila* facilitating the recognition of pathogens, and the subsequent activation of various immune modules, notably phagocytosis or the Toll pathway. These findings may shed light on the evolution of microbe recognition and activation of immune responses, particularly the crosstalk between TLR signalling and complement cascades in mammalian immunity [60].

## Additional files

Additional file 1: Figure S1. A. Molecular confirmation of the *TEPq* ^*Δ*^ mutant. Expression of *TEP1, 2, 3* and *4* was monitored by qRT-PCR in the *w* ^*1118*^ and *TEPq* ^*Δ*^ flies after bacterial challenge. Female flies were pricked in the thorax with a needle dipped in a concentrated mixed culture of *E. faecalis* (OD 0.5) and *E. carotovora* (OD 200). Expression of *TEP* genes was measured 6 h post-infection using primers TEP1F, TEP1R, TEP2F, TEP2R, TEP3F, TEP3R, TEP4F and TEP4R (for sequences see Additional file 3: Table S1). The expression of all four genes was strongly reduced in the *TEPq* ^*Δ*^ line as compared to *w* ^*1118*^, confirming the *TEPq* ^*Δ*^ genotype. Furthermore, we confirmed the presence of the expected transposon insertion (see Fig. 1b) in the *TEPq* ^*Δ*^ mutant in the respective loci by PCR. Insertion of the MiMiC element in the *Tep1* locus was confirmed using primers dTEP1F and dTEP1R. An approximately 700-bp fragment was amplified in the *TEPq* ^*Δ*^ mutant but not in the control wild-type line. Insertion of the EP element in the *Tep4* locus was confirmed using primers dTEP4F and dTEP4R. An approximately 330-bp fragment was amplified in the *TEPq* ^*Δ*^ mutant but not in the control wild-type line. Presence of a deletion due to the flippase excision of the genomic region between the flippase recognition target (*FRT*) sites of the two XP elements in the *Tep*2 and *Tep3* loci was confirmed using primers dTEP2&3 F and dTEP2&3R. No amplification was observed in the *TEPq* ^*Δ*^ mutant; an approximately 630-bp fragment was amplified in the control wild-type line. Sequences of all primers used are indicated in Additional file 3: Table S1. B. Viability of the *TEPq* ^*Δ*^ line. *TEPq* ^*Δ*^/CyO males were crossed to *TEPq* ^*Δ*^ homozygous females, and the ratio of homozygous versus heterozygous flies was counted. A significantly higher number of *TEPq* ^*Δ*^/CyO offspring was observed (*P* < 0.001), suggesting a reduced viability of the *TEPq* ^*Δ*^ homozygous line. C. The level of *Socs36E* expression 2 h after clean injury is similar in the *TEPq* ^*Δ*^ flies compared to wild-type flies (Mann-Whitney test two-sided, *P* > 0.05). Shown is the level of *Socs36E* expression normalised to *RpL32* in unchallenged (*UC*) flies and in flies collected 2 h after being pricked in the thorax with a clean needle. D. Flies were pricked in the thorax with a clean needle, and the level of melanisation at the wound site, estimated by the size of the melanin spot, was examined 3 h later. *TEPq* ^*Δ*^ flies showed a normal rate of surface melanisation of the wound site. E. Melanisation in flies after infection with *P. luminescens*. No statistically significant difference was observed between the *TEPq* ^*Δ*^ *, TEP4* ^*Δ*^ and wild-type flies (chi-square test, *P* = 0.0681)*. (PDF 526 kb)*

Additional file 2: Figure S2. A. Survival of *TEPq* ^*Δ*^ flies to septic injury with *B. bassiana*. This *TEPq* ^*Δ*^ fly line was generated by directly recombining previously described mutations affecting *TEP1, TEP2,3* and *TEP4* without any backcross into the *w* ^*1118*^ genetic background. Male flies were pricked in the thorax with a needle dipped in a concentrated fungal spore suspension. Despite their distinct genetic background, *TEPq* ^*Δ*^ flies were more susceptible to infection than wild-type flies from two different backgrounds (*w* ^*1118*^ and OregonR) (*P* < 0.001 for both *TEPq* ^*Δ*^ compared to *w* ^*1118*^ and *TEPq* ^*Δ*^ compared to OregonR (*Or*) flies. B. Survival of individual *TEP* mutants, double *TEP* mutants and the *TEPq* ^*Δ*^ flies (all in the *w* ^*1118*^ genetic background) to natural infection with *B. bassiana*. Male flies were covered with spores. The *TEP1* ^*Δ*^, *TEP1,4* ^*Δ*^, *TEP2,3* ^*Δ*^ and *TEPq* ^*Δ*^ showed statistically significantly higher susceptibility than the control *w* ^*1118*^ flies. Data were analysed by log-rank test. Shown are representative experiments of two independent repeats. *x*-axis: time post-infection in days; *y*-axis: percentage of living flies. (PDF 420 kb)

Additional file 3: Table S1. List of primers used in this study. (XLSX 9 kb)

Additional file 4: Table S2. Additional file containing raw data generated in this study. (XLSX 40 kb)

## Abbreviations

Dpt: Diptericin
Drs: Drosomycin
PBS: phosphate-buffered saline pH 7.4
