# Supplementary figures and images for: Thioester-containing proteins regulate the Toll pathway and play a role in Drosophila defence against microbial pathogens and parasitoid wasps
Source: BMC Biol. 2017 Sep 5;15:79. doi: 10.1186/s12915-017-0408-0 (PMC5584532; doi:10.1186/s12915-017-0408-0)

Figure S1:

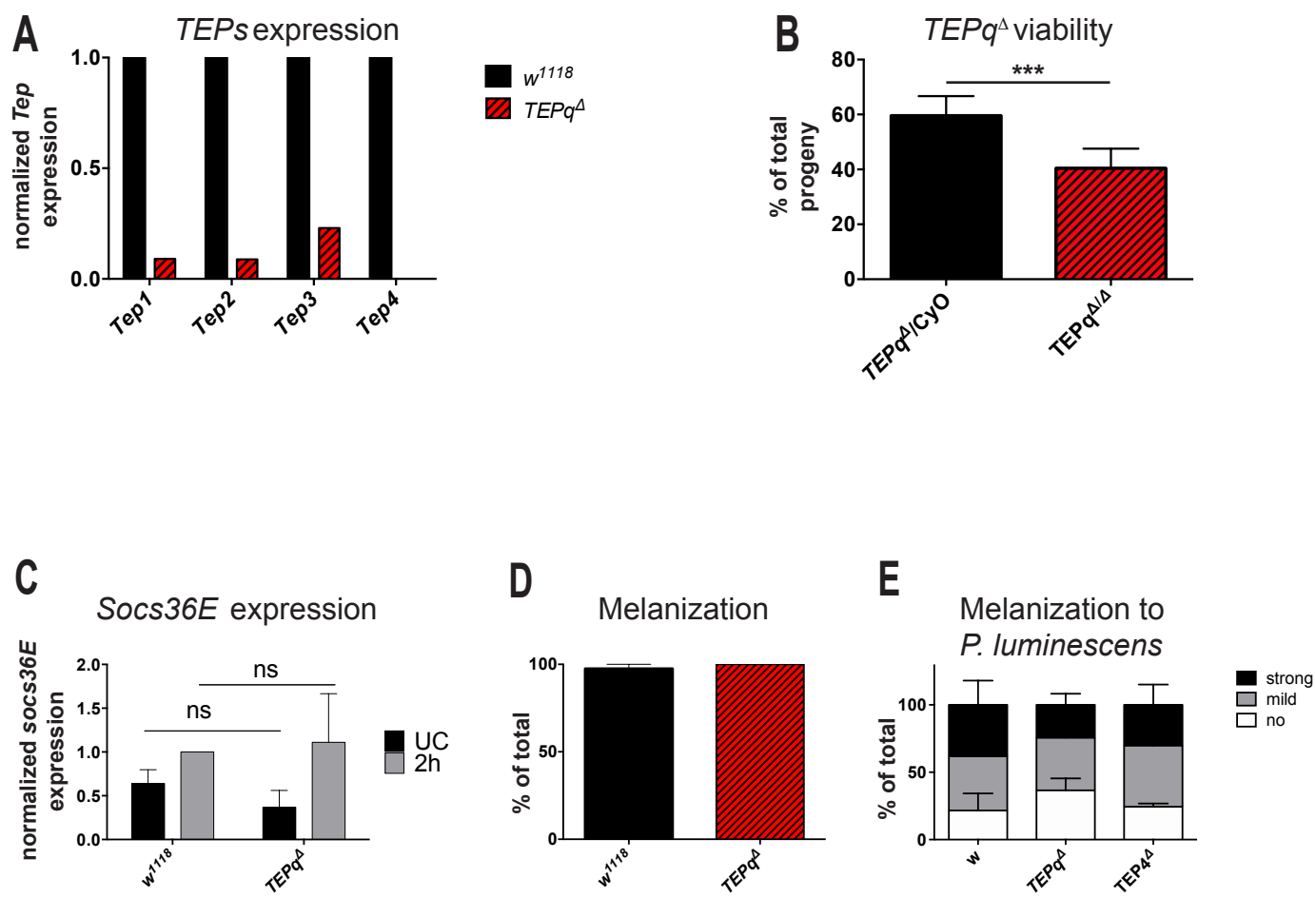

Supplement: Supplementary file 1 — A. Molecular confirmation of the TEPq Δ mutant. Expression of TEP1, 2, 3 and 4 was monitored by qRT-PCR in the w 1118 and TEPq Δ flies after bacterial challenge. Female flies were pricked in the thorax with a needle dipped in a concentrated mixed culture of E. faecalis (OD 0.5) and E. carotovora (OD 200). Expression of TEP genes was measured 6 h post-infection using primers TEP1F, TEP1R, TEP2F, TEP2R, TEP3F, TEP3R, TEP4F and TEP4R (for sequences see Additional file 3: Table S1). The expression of all four genes was strongly reduced in the TEPq Δ line as compared to w 1118, confirming the TEPq Δ genotype. Furthermore, we confirmed the presence of the expected transposon insertion (see Fig. 1b) in the TEPq Δ mutant in the respective loci by PCR. Insertion of the MiMiC element in the Tep1 locus was confirmed using primers dTEP1F and dTEP1R. An approximately 700-bp fragment was amplified in the TEPq Δ mutant but not in the control wild-type line. Insertion of the EP element in the Tep4 locus was confirmed using primers dTEP4F and dTEP4R. An approximately 330-bp fragment was amplified in the TEPq Δ mutant but not in the control wild-type line. Presence of a deletion due to the flippase excision of the genomic region between the flippase recognition target (FRT) sites of the two XP elements in the Tep2 and Tep3 loci was confirmed using primers dTEP2&3 F and dTEP2&3R. No amplification was observed in the TEPq Δ mutant; an approximately 630-bp fragment was amplified in the control wild-type line. Sequences of all primers used are indicated in Additional file 3: Table S1. B. Viability of the TEPq Δ line. TEPq Δ/CyO males were crossed to TEPq Δ homozygous females, and the ratio of homozygous versus heterozygous flies was counted. A significantly higher number of TEPq Δ/CyO offspring was observed (P < 0.001), suggesting a reduced viability of the TEPq Δ homozygous line. C. The level of Socs36E expression 2 h after clean injury is similar in the TEPq Δ flies compared to wild-ty [file 12915_2017_408_MOESM1_ESM.pdf]

**Figure S2:**

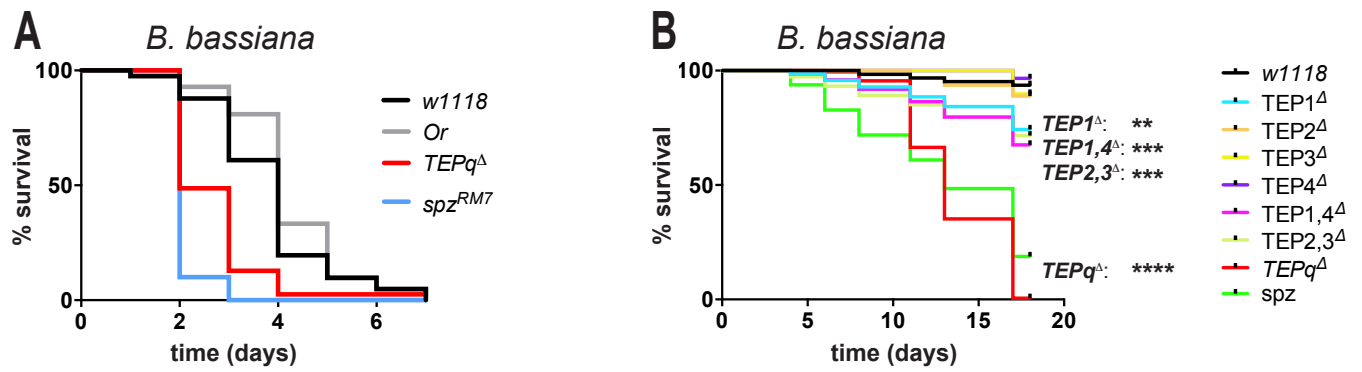

Supplement: Supplementary file 2 — A. Survival of TEPq Δ flies to septic injury with B. bassiana. This TEPq Δ fly line was generated by directly recombining previously described mutations affecting TEP1, TEP2,3 and TEP4 without any backcross into the w 1118 genetic background. Male flies were pricked in the thorax with a needle dipped in a concentrated fungal spore suspension. Despite their distinct genetic background, TEPq Δ flies were more susceptible to infection than wild-type flies from two different backgrounds (w 1118 and OregonR) (P < 0.001 for both TEPq Δ compared to w 1118 and TEPq Δ compared to OregonR (Or) flies. B. Survival of individual TEP mutants, double TEP mutants and the TEPq Δ flies (all in the w 1118 genetic background) to natural infection with B. bassiana. Male flies were covered with spores. The TEP1 Δ, TEP1,4 Δ, TEP2,3 Δ and TEPq Δ showed statistically significantly higher susceptibility than the control w 1118 flies. Data were analysed by log-rank test. Shown are representative experiments of two independent repeats. x-axis: time post-infection in days; y-axis: percentage of living flies. (PDF 420 kb) [file 12915_2017_408_MOESM2_ESM.pdf]
